# Replicates and repeats

**DOI:** 10.1186/s12915-016-0254-5

**Published:** 2016-04-07

**Authors:** Graham Bell

**Affiliations:** BMC Biology, BioMed Central, 236 Gray’s Inn Road, London, WC1X 8HB UK

## Abstract

The authors of this paper were interested to see whether the expression of three proteins (A, B and C) was altered in a knockout mouse model of a gene encoding Protein X. Each experiment measured the expression of a different protein and the results seem to show a clear increase in protein expression in the mutant mouse compared with the control. The results are reported as statistically significant, with impressive ‘n’ values in each condition: the authors say that *n* = 7 in each case. They confidently conclude that the level of each of the three proteins is increased in the knockout mutant.

## Comment

Repeating an experiment to be confident that an observed effect represents a real phenomenon is key in biology and the reproducibility (or lack thereof) of research has garnered much attention recently. One important factor to consider (and report) is whether the replicates for each experiment are biological replicates or technical replicates. Broadly speaking, biological replicates are biologically distinct samples (e.g. the same type of organism treated or grown in the same conditions), which show biological variation; technical replicates are repeated measurements of a sample, which show variation of the measuring equipment and protocols.

**Fig. 1:**
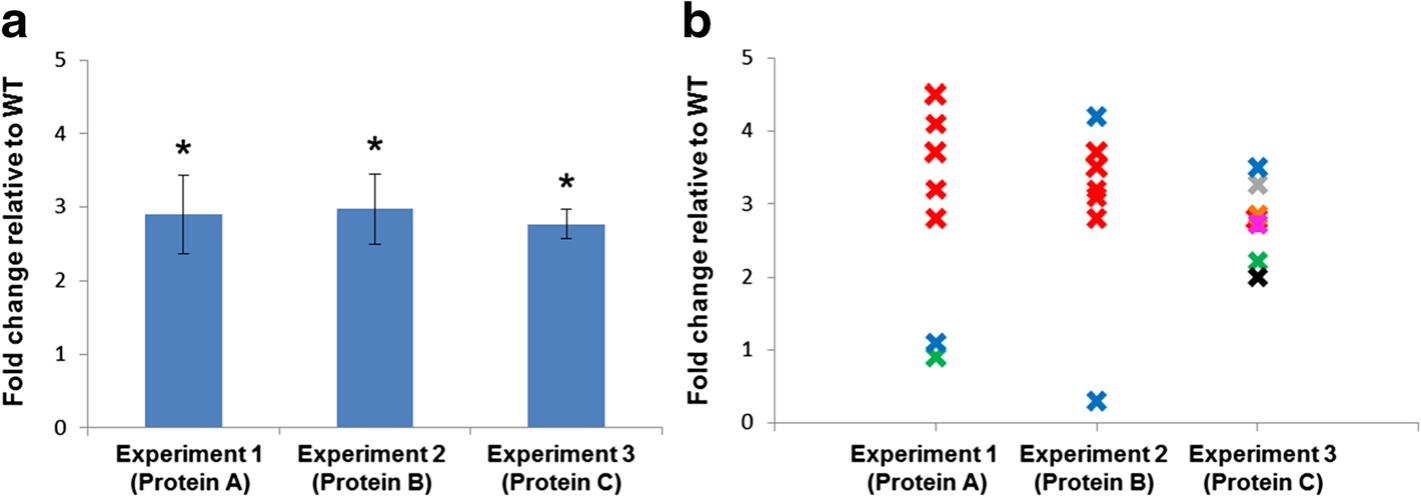
**a** Increased expression of Proteins A, B and C in *geneX* knockout mice relative to wild type (*WT*). *n* = 7 for each experiment. **b** The same data as in **a**, but with individual data points plotted. Different colours refer to different mice. *P < 0.05, two-tailed t-test. Error bars show SD

Figure 1a shows the results of three experiments, each measuring the increase in expression of a protein in a knockout mouse model relative to the wild type, and the authors say in each case that “*n* = 7”. However, only Experiment 3 provides statistically significant support for their conclusions because of the inappropriate way the data were handled in Experiments 1 and 2.

In Fig. 1b, the data values for each of the seven “n” values are plotted in each experiment. Different colours represent different biological replicates (i.e. different mice). Experiment 3 used seven different mice, measured once each. Experiment 1 used three mice but measured one of them five times, resulting in five technical replicates (red crosses). This skews the calculated mean heavily in favour of results from that mouse. If those technical replicates were combined into a single value, and the results from each mouse were given equal weighting, the overall result would no longer be statistically significant, since mice #2 and #3 (blue and green crosses) show no real change compared with the wild type.

In Experiment 2, although seven values are given, these come from only two mice (two biological replicates), with each measured more than once. The n value should therefore be reported as 2, not 7, and *p* values shouldn’t be given. The authors should also be concerned that the technical replicates for mouse #2 (blue crosses) are very different, suggesting that their equipment was faulty.

